# Assessment of local and systemic signature of eosinophilic esophagitis (EoE) in children through multi-omics approaches

**DOI:** 10.3389/fimmu.2023.1108895

**Published:** 2023-03-15

**Authors:** Karine Adel-Patient, Florence Campeotto, Marta Grauso, Blanche Guillon, Marco Moroldo, Eric Venot, Céline Dietrich, François Machavoine, Florence A. Castelli, François Fenaille, Thierry Jo Molina, Patrick Barbet, Christophe Delacourt, Maria Leite-de-Moraes, Guillaume Lezmi

**Affiliations:** ^1^ Université Paris-Saclay, CEA, INRAE, Département Médicaments et Technologies pour la Santé (DMTS), Gif-sur-Yvette, France; ^2^ AP-HP, Hôpital Necker-Enfants Malades, Service de Gastro-Entérologie et Nutrition Pédiatriques, Paris, France; ^3^ Université de Paris Cité, INSERM UMR1139, Laboratoire de Microbiologie, Faculté de Pharmacie, Paris, France; ^4^ Université Paris Saclay, INRAE, AgroParisTech, GABI, Jouy-en-Josas, France; ^5^ Université Paris Cité, CNRS UMR 8253, Inserm UMR 1151, Institut Necker Enfants Malades, Equipe Immunorégulation et Immunopathologie, Paris, France; ^6^ Université de Paris, UMRS 1138, INSERM, Sorbonne Paris-Cité, Paris, France; ^7^ AP-HP, Centre-Université de Paris, hôpital Necker-Enfant-Malades, Service d'Anatomie et Cytologie Pathologiques, Paris, France; ^8^ AP-HP, Hôpital Necker-Enfants Malades, Service de Pneumologie et Allergologie Pédiatriques, Paris, France

**Keywords:** children, food allergy, Eosinophilic oesophagitis, immune response, multi-omics signature, transcriptomics, metabolomics

## Abstract

**Background:**

Eosinophilic oesophagitis (EoE) is a chronic food allergic disorder limited to oesophageal mucosa whose pathogenesis is still only partially understood. Moreover, its diagnosis and follow-up need repeated endoscopies due to absence of non-invasive validated biomarkers. In the present study, we aimed to deeply describe local immunological and molecular components of EoE in well-phenotyped children, and to identify potential circulating EoE-biomarkers.

**Methods:**

Blood and oesophageal biopsies were collected simultaneously from French children with EoE (n=17) and from control subjects (n=15). Untargeted transcriptomics analysis was performed on mRNA extracted from biopsies using microarrays. In parallel, we performed a comprehensive analysis of immune components on both cellular and soluble extracts obtained from both biopsies and blood, using flow cytometry. Finally, we performed non-targeted plasma metabolomics using liquid chromatography coupled to high-resolution mass spectrometry (LC-HRMS). Uni/multivariate supervised and non-supervised statistical analyses were then conducted to identify significant and discriminant components associated with EoE within local and/or systemic transcriptomics, immunologic and metabolomics datasets. As a proof of concept, we conducted multi-omics data integration to identify a plasmatic signature of EoE.

**Results:**

French children with EoE shared the same transcriptomic signature as US patients. Network visualization of differentially expressed (DE) genes highlighted the major dysregulation of innate and adaptive immune processes, but also of pathways involved in epithelial cells and barrier functions, and in perception of chemical stimuli. Immune analysis of biopsies highlighted EoE is associated with dysregulation of both type (T) 1, T2 and T3 innate and adaptive immunity, in a highly inflammatory milieu. Although an immune signature of EoE was found in blood, untargeted metabolomics more efficiently discriminated children with EoE from control subjects, with dysregulation of vitamin B6 and various amino acids metabolisms. Multi-blocks integration suggested that an EoE plasma signature may be identified by combining metabolomics and cytokines datasets.

**Conclusions:**

Our study strengthens the evidence that EoE results from alterations of the oesophageal epithelium associated with altered immune responses far beyond a simplistic T2 dysregulation. As a proof of concept, combining metabolomics and cytokines data may provide a set of potential plasma biomarkers for EoE diagnosis, which needs to be confirmed on a larger and independent cohort.

## 1 Introduction

Eosinophilic oesophagitis (EoE) is a chronic and local type-2 (T2) inflammatory disorder. This food allergy is characterized by symptoms of oesophageal dysfunction including dysphagia, food impaction, heartburn, chest pain, and vomiting. It may lead to food refusal and persistent reflux in young children. The diagnosis of EoE and its monitoring require endoscopy to assess macroscopic changes of the oesophagus and oesophageal eosinophilia (1–4).

EoE is now considered to be a heterogeneous condition encompassing various endotypes (5). An oesophageal EoE transcriptomic signature has been identified for US patients (6, 7), and an EoE diagnostic panel (EDP) based on a 94-gene quantitative PCR-array was described to distinguish children or adults with EoE from age-matched controls (5, 8). At the protein level, increased concentrations of Type 2 cytokines such as eotaxin-3 (CCL26) (6) and various antibody isotypes (9) have been found in oesophageal biopsies. Other studies showed the presence of mast cells, B and T lymphocytes, and type 2 innate lymphoid cells (ILC2) within the oesophageal mucosa (6, 10–13). However, these results were obtained mostly from targeted analysis and few studies have analysed the immune components using more integrated and global approaches.

Importantly, non-invasive biomarkers of EoE have not yet been identified (14), and no immune signature specific for EoE has been described in blood (15). Interestingly, a preliminary study performed targeted metabolomics by analysing 48 metabolites in plasma from a small cohort (16). However, untargeted metabolomics, i.e. the comprehensive analysis of all the low molecular weight components (<1000-1500 Da), may be a more promising approach to assess altered pathways that contribute to or result from complex diseases, including food allergies (17), and to highlight relevant biomarker candidates. Using such an approach, we identified a metabolomics signature in plasma from children with another locally restricted food allergy, i.e., food-protein induced enterocolitis syndrome (FPIES) (18).

In this study, we then aimed to deeply describe immunological and molecular components of EoE in well-phenotyped French children. We defined their EoE transcriptomic and immune signatures in biopsies, and performed a comprehensive description of EoE-related immune constituents and metabolome in blood. Multi-block dataset integration was then performed, allowing envisaging the identification of an EoE signature in plasma.

## 2 Materials and methods

### 2.1 Ethical statement

The study was approved by the local ethics committee of Necker Hospital CPP Ile de France II (n°2016-06-03) and informed signed consent were obtained from all parents.

### 2.2 Patient recruitment

Children requiring upper digestive endoscopy for EoE or to explore chronic gastroesophageal reflux disease (GERD) or abdominal pain were recruited at Necker-Enfants Malades Hospital (Paris, France). Biopsies were collected from the upper, middle, and lower third part of the oesophagus for the analysis of oesophageal inflammation; eosinophil counts were assessed in each part of the oesophagus in the department of pathology. In children with oesophageal dysfunction, the diagnosis of EoE was confirmed by the presence of >15 eosinophils per high-power field (HPF) in at least one part of the oesophagus (19). Control subjects (CT) presented with symptoms of GERD, abdominal pain or dysphagia, and had <15 eosinophils/HPF.

In parallel, a biopsy was placed in RNA*later*™ (Invitrogen, ThermoFisher Scientific, France) and transferred to -80°C until transcriptomic analysis. Another biopsy was placed in tissue storage solution (Miltenyi Biotec GmbH, Germany), and maintained at 4°C. They were analysed within 24 h for immunophenotyping and for the assessment of local cytokine/antibody concentrations. Blood samples were collected in parallel to the biopsy during anaesthesia (Vacutainer® sodium heparin tubes, BD, Le Pont de Claix, France). Blood samples were maintained at room temperature and processed within 4 h for immunophenotyping and analysis of plasma cytokines, antibodies and metabolome. All samples were anonymised and blinded for case/control status until final statistical analysis.

### 2.3 Transcriptomic analysis

Total RNA was purified using the AllPrep® DNA/RNA Micro kit (Qiagen S.A.S., Hilden, Germany), following the provider’s recommendations. The RNA concentration and 260/230 nm and 260/280 nm ratio were determined by spectrometry (Nanodrop, Thermo Fisher Scientific, Waltham, MA, US). The RNA integrity number (RIN) was determined using an Agilent 2100 Bioanalyzer (Agilent Technologies, Santa Clara, CA) and an RNA 6000 Nano Chip. Of the RNA extracted from the 31 available biopsies, 28 samples (15 EoE and 13 CT) showed good RNA quantity and integrity (RIN ≥ 6.8). These 28 samples were used to hybridise SurePrint G3 Human Gene Expression v3 8x60K microarrays (AMADID 072363, Agilent Technologies, Santa Clara, CA, US) at the @BRIDGe genomics core facility (INRAE, Jouy-en-Josas, France http://abridge.inrae.fr/) (untargeted transcriptomics). Cyanine-3 (Cy3)-labelled cRNA was prepared using 50 ng of total RNA (One-Color Low Input Quick Amp Labeling kit, Agilent Technologies). Specific activities and cRNA yields were determined (Nanodrop, Thermo Fisher Scientific). For each sample, 600 ng of Cy3-labeled cRNA (specific activity > 9.0 pmol Cy3/µg of cRNA) was fragmented at 60°C for 30 min and used for hybridization (17 h at 65°C under rotation in a hybridization oven, Agilent Technologies). After hybridization, microarrays were washed and the slides scanned (G2565CA Scanner System, Agilent Technologies; resolution of 3 µm and dynamic range of 20 bits). The resulting .tiff images were analysed using Feature Extraction Software version 12.0.3.2 (Agilent Technologies, GE1_1200_Jun14 protocol).

Probe intensities were background-corrected using the “normexp” method, log2 scaled, and quantile normalized using the limma R package (version 3.1.42.2) in the R environment (version 3.6.1) (20). The probes of the lowest quartile were filtered out, the controls discarded, and the probes corresponding to genes summarized. The obtained expression matrix was processed (arrayQualityMetrics R package, version 3.42.0) (21) and subsequently used to perform a scaled PCA analysis for quality assessment (FactoMineR R package, version 2.4 (22);). Differentially expressed (DE) genes were identified using the limma R package; a linear model was fitted for each gene comparing EoE patients to CT, with a threshold of 0.05 for Benjamini-Hochberg-corrected *p*-values. Of note, neither additional DE genes nor clustering were found when integrating proton pump inhibitor (PPI) medication into the linear model; we thus considered that EoE transcriptomic signature was not affected by PPI use in the subsequent analysis. Among the transcriptomic data obtained from 28 biopsies, non-supervised PCA detected four outliers, corresponding to two children with EoE and two CT (not shown). Three were characterized by a lower RIN value (< 7.8, Med_RIN_ of all samples = 8.95 [7.3-9.7]), suggesting that RNA quality may have affected the results. Actually, of the 26,803 unique genes and 30,606 unique lncRNAs represented on the microarrays, analysis of the 28 samples evidenced only 161 differentially expressed (DE) genes associated with EoE, whereas 943 DE genes were found when excluding the four outliers, which were therefore excluded for further analysis that will include 13 EoE and 11 CT. The 50 highest and the 50 lowest DE genes were selected from the expression matrix to generate a heatmap plot; data were centred using the scale R function (stats R package version 3.6.2). The results obtained for DE genes were functionally analysed using various approaches. First, the DE gene lists were analysed using Ingenuity Pathways Analysis 01.08 (IPA, www.ingenuity.com, March 2021 Release) to identify canonical pathways. Only pathways with a -log(p-value) > 2.5 and including at least 10 genes were considered. The second analysis used functionally organized gene ontology/pathway term networks [ClueGO 2.5.7 (23)]: a two-sided test was used to highlight the level of enrichment in functional terms among the DE genes. Significance was set to an adjusted *p*-value of 0.05, the ‘GO fusion’ option used, and the k-score fixed at 0.4 and the global/local parameter at the sixth level. Two ontologies were selected, ‘Homo sapiens GO Biological Processes’ and ‘Homo sapiens KEGG’ (08/05/2020 updates). The third approach used was gene set enrichment analysis [GSEA, http://software.broadinstitute.org/gsea/index.jsp (24)]. The expression matrices including all analysed transcripts were analysed using the ‘KEGG C2 curated gene set’ from the MsigDB Database 7.2 (2.500 permutations, “gene_set” option). Finally, a literature-based meta-analytical enrichment test was carried out. The gene signature published by Blanchard and collaborators (6) was retrieved and the Affymetrix probe sets converted into the corresponding gene symbols using the ‘db2db’ conversion tool (https://biodbnet-abcc.ncifcrf.gov/db/db2db.php). A final set of 406 unique gene symbols was obtained. In parallel, the genes we found in the DE gene lists were filtered by removing all genes that lacked an annotated gene symbol and of which the name started with the prefixes ‘ENST’,’ A_’, ‘LINC’, ‘LINC-ROR’, ‘lnc’, ‘LNCAROD’. These genes correspond to poorly annotated genomic regions or long non-coding RNAs and were not found in the list published (6). A final list of 300 DE genes was obtained. The levels of enrichment were assessed using Fisher’s exact test and the correlations among the log fold change (logFC) values visualized using a dot plot (grid R package, version 3.6.2).

### 2.4 Blood collection; PBMC and plasma separation

Specific IgE to common food (cow’s milk, egg, wheat, legumes, fish, and nuts) and respiratory (house dust mites, grass, birch, and cat and dog dander) allergens were determined using plasma from one blood sample (ImmunoCap, Phadia). In parallel, blood samples were diluted 1/2 in AIM-V® serum-free medium (ThermoFisher Scientific) and peripheral blood mononuclear cells (PBMCs) and plasma were obtained (Histopaque®-1077, Sigma Aldrich, St Louis, USA). Plasma was aliquoted and stored at -80°C until antibody/cytokine and metabolomics analysis. PBMCs were suspended in 5 mL AIM-V® medium supplemented with 1% autologous plasma and maintained at 4°C until flow cytometry analysis. Plasma and PBMC samples were avalaible for the whole cohort.

### 2.5 Lymphoid cell isolation from oesophageal biopsies

Biopsies were washed twice in RPMI-1640 medium containing 10 mM Hepes and then digested using 42 µg/mL Liberase™S and 1666 U/mL DNase I (all from Roche Diagnostics GmbH) for 45 min at 37°C, followed by mechanical dissociation (gentleMACS™ Dissociator, Miltenyi). The suspension was filtered (70 µm) and centrifuged (1000 x *g*, 5 min, 4°C). The cell pellet was treated as described for PBMC. The supernatant was collected and supplemented with protease inhibitor cocktail (100x, Sigma), then aliquoted and stored at -80°C until antibody/cytokine analysis. Supernatant samples were not available for 3 CT and 2 EoE patients.

### 2.6 Flow cytometry analysis

Subtypes of helper T cells (Th) and innate lymphoid cells (ILC) were analysed in blood and biopsies as previously described (25). Activated mucosal-associated invariant T (MAIT) and invariant natural killer T (iNKT) cells were analysed in blood as previously described (26). Within circulating FSC^low^CD45^+^ live singlet cells, cells analysed and considered in statistical analysis were: CD4^+^ T cells (lin^+^CD4^+^) and, within CD4^+^, Th1 (t-bet^+^) and activated Th1 (IFNγ^+^), Th2 (GATA-3^+^) and activated Th2 (IL-13^+^), Th17 (RORγt^+^) and activated Th17 (IL-22^+^); CD8^+^ T cells and within CD8^+^ cells those positive for IFNγ, IL-4, IL-13 or IL-17; total ILC (FSC^low^CD45^+^, live singlet cells, lin^-^CD127^+^), then ILC1 (t-bet^+^) and activated ILC1 (IFNγ^+^), ILC2 (GATA-3^+^) and activated ILC2 (IL-13^+^), ILC3 (RORγt^+^) and activated ILC3 (IL-22^+^); total iNKT cells (identified within CD3^+^ cells using CD1-PBS57 tetramers), then iNKT positive for IFNγ, IL-4, IL-13 or IL-17; Vδ2^+^ cells within CD3^+^iNKT^-^ and corresponding IFNγ^+^, IL-4^+^, IL-13^+^ or IL-17^+^ cells; total MAIT cells (CD3^+^TCRVα7.2^+^CD161^+^) and MAIT positive for IFNγ, IL-4, IL-13 or IL-17. Within FSC^low^CD45^+^ live singlet cells in biopsies, cells analysed and considered in statistical analysis were CD4^+^ cells, Th1 and activated Th1, Th2 and activated Th2, Th17 and activated Th17, total ILC, then ILC1 and activated ILC1, ILC2 and activated ILC2, ILC3 and activated ILC3. In total, forty cell subtypes (T, ILC, MAIT, iNKT) and fourteen cell subtypes (Th, ILC) were analysed within PBMC and biopsies, respectively. Due to high cell mortality or insufficient cell numbers, data were excluded for three CT and three EoE patients for Th cells, and for three CT and one EoE patient for ILC (overlapping with the Th missing data).

### 2.7 Analysis of cytokines and antibodies in biopsy supernatants and plasma

Forty chemokines (Bio-Plex_Pro™ Human chemokine assays, BioRad), 37 inflammation markers (Bio-Plex_Pro™ Human Inflammation Panel 1, BioRad), six Th17-associated cytokines (Bio-Plex_Pro™ Human Th17 cytokines assays), and IL-5 and IL-13 (singlex from Bio-Plex_Pro™ Human cytokine screening panel) were analysed in oesophageal supernatants and plasma samples following the manufacturer’s recommendations. Due to some redundancy between the kits, 80 immune soluble constituents were analysed for each sample: APRIL/TNFSF13, BAFF/TNFSF13B, sCD30/TNFRSF8, sCD163, Chitinase 3-like 1, CCL21 (6Ckine), CXCL13 (BCA-1), CCL27 (CTACK), CXCL25 (ENA-78), CCL11 (Eotaxin), CCL24 (Eotaxin-2), CCL26 (Eotaxin-3), CX3CL1 (Fractalkine), CXCL6 (GCP-2), GM-CSF, CXCL1 (Gro-α), CXCL2 (Gro-β), CCL1 (I-309), gp130/sIL-6Rβ, sIL-6Rα, IFNα2, IFNβ, IFNγ, IL-1β, IL-2, IL-4, IL-5, IL-6, IL-8 (CXCL8), IL-10, IL-11, IL-12p40, IL-12p70, IL-13, IL-16, IL-17A, IL-17F, IL-19, IL-20, IL-21, IL-22, IL-26, IL-27 (p28), IL-28A (IFN-λ2), IL-29 (IFN-λ1), IL-32, IL-33, IL-34, IL-35, CXCL10 (IP-10), CXCL11 (I-TAC), CCL2 (MCP-1), CCL8 (MCP-2), CCL7 (MCP-3), CCL13 (MCP4), CCL22 (MDC), MIF, CXCL9 (MIG), CCL3 (MIP-1α), CCL15 (MIP-1δ), CCL20 (MIP-3α), CCL19 (MIP-3β), CCL23 (MPIF-1), CXCL16 (SCYB16), CXCL12 (SDF-1α+β), CCL17 (TARC), CCL25 (TECK), LIGHT/TNFSF14, MMP-1, MMP-2, MMP-3, Osteocalcin, Osteopontin (OPN), Pentraxin-3, sCD40L, sTNF-R1, sTNF-R2, TLSP, TNFα, and TWEAK/TNFSF12. Overall, 79 and 46 cytokines/chemokines were significantly quantified (i.e. were superior to limit of quantification) in plasma and biopsy supernatants, respectively.

Total IgG1, IgG2, IgG3, IgG4, IgA, IgE and IgM were analysed in plasma and biopsy supernatants, using Bio-Plex Pro™ Human isotyping panel (BioRad). IgM were not detected in the biopsy supernatants. Total IgE were also assayed using an in-house specific immunoassay, with comparable results (not shown) (27).

### 2.8 Metabolomics analysis

Untargeted metabolomics was performed after the extraction of plasma metabolites thanks to methanol-assisted protein precipitation as described before (28). Supernatants were analysed by liquid chromatography (LC) using two complementary conditions (ultra-high-performance LC, UHPLC; Hypersil GOLD C18 and high-performance LC, HPLC, Sequant ZIC-pHILIC; Dionex Ultimate chromatographic system) designated hereafter as C18 and HILIC. LC was directly coupled to a mass spectrometer (Exactive, Orbitrap; Thermo Fisher Scientific, Courtaboeuf, France) fitted with an electrospray source operated in the positive (C18) and negative (HILIC) ion modes. The software interface was Xcalibur (version 2.1) (Thermo Fisher Scientific, Courtaboeuf, France).

Data processing and statistical analyses were performed using the open-source web-based platform workflow4metabolomics (W4M: http://workflow4metabolomics.org (29),). Automatic peak detection and integration were performed using the matched filter algorithm in the W4M pre-processing package (including XCMS software tool). Features generated from XCMS were filtered according to the following criteria: (i) correlation between QC dilution factors and areas of chromatographic peaks (> 0.7), (ii) coefficient of variation of chromatographic peak areas of QC samples (< 30%), and (iii) ratio of chromatographic peak areas of biological to blank samples (> 3).

Feature annotation was performed at level 2 according to Metabolomics Standards Initiative (30) using our in-house spectral database listing authentic chemical standards analysed under the same analytical conditions than samples (28, 31). To be annotated, ions had to match accurate measured mass (considering a ± 10 ppm mass tolerance) and retention time (considering a ± 0.3 min tolerance for the annotation of the C18 dataset, and a ± 0.8 min tolerance for the annotation of the HILIC data set). C13 isotopic pattern and integration quality of the extracted peak were checked on raw data using Xcalibur and TraceFinder softwares (Thermo Fisher Scientific, Courtaboeuf, France).

### 2.9 Statistical analysis

The data for soluble and cellular immune constituents and from untargeted metabolomics analysis were not normally distributed. To obtain an overview of the variables and individuals and identify potential outliers (none identified), we first performed a descriptive analysis (principal component analysis, PCA) of all immune constituents or annotated metabolites after mean centring the variables and scaling them to unit variance. In certain analyses, we performed non-supervised classification based on the Euclidian distance (Ward’s Method, Ascendant Hierarchical Clustering - AHC) to assess the natural clustering of samples based on all measured constituents. Data modelling was then performed using supervised partial least square-discriminant analysis (PLS-DA), with the EoE status as the explicative variable. Successful construction of the models indicates that it is possible to classify the EoE patients vs CT based on all measured immune constituents/metabolites. The robustness of the models is evaluated based on the R²X (explained variance) and R²Y (capability of prediction) scores. Such models allow the identification of “discriminant variables”, i.e. constituents that mostly participate in constructing the models and then mostly support the differences between the patient groups. These constituents are identified based on model-calculated variable-important-in-projection values (VIP > 1). In parallel, we performed pairwise univariate comparisons of each immune constituent using the non-parametric Mann-Whitney test and the corresponding P values were obtained. As a final step, the measured constituents showing a VIP > 1 and a P value < 0.05 were selected to identify the sets of immune constituents or metabolites that discriminated the most significantly between children with EoE and controls.

### 2.10 Data integration

As a proof of concept, expression profiles in biopsies and plasma levels of cytokines, immunoglobulins and annotated metabolites were integrated using the DIABLO framework of the mixOmics R package (version 6.14.1). The relationships among these four datasets were studied by including EoE as a categorical variable. This analysis considered only the 10 EoE patients and 10 controls identified in our initial transcriptomic analysis, i.e. evidencing clear EoE transcriptomics signature and who had no missing data in the other blocks. All data sets underwent a number of pre-processing steps: for DE genes, an expression matrix was filtered out by retaining only the genes that showed FC>|2.0|, which led to a matrix of 341 DE genes; plasma cytokine data were log2 scaled; plasma immunoglobulin data and metabolome data were log10 scaled. A first DIABLO model was fitted without variable selection to assess the global performance of the model itself. The function ‘perf’ was run with 10-fold cross validation repeated 10 times and the obtained ‘diagnostic plot’ indicated that three components were optimal for the final model. For the fine-tuning of the final model, a grid of ‘keepX’ values was defined as follows: (1) DE genes: 50, 40, 30; (2) plasma cytokines: 20, 15, 10; (3) plasma immunoglobulins: 5, 4, 3; and (4) plasma metabolites: 30, 25, 20. Then, the ‘tune’ function was run with 10-fold cross validation but for only one repetition. The final model was fitted by selecting three components and by considering the results of the previous fine-tuning step. The results were visualized by drawing a ‘DIABLO plot’, an ‘Individual plot’, and a ‘Circos plot’. For the Circos plot, only interactions with *r* ≥|0.70| were visualized.

## 3 Results

### 3.1 Patients

Seventeen children with EoE and 15 CT subjects were included (**Table 1**). Ten children with EoE and eight CT subjects were not under PPI medication at inclusion. Children with EoE were characterized by higher level of total IgE and were more frequently sensitized to common food and respiratory allergens. Food impaction was the only symptom observed more frequently in children with EoE than in CT subjects.

**Table 1:** General characteristics of the population.

	Patients		Control subjects	p value
N	17		15	
Age (years)	10.9 [7.7 - 14.3]		7.7 [4.70-11.6]	0.06
BMI, kg.m^-2^	16.33 [14.37-20.28]		15.51 [12.00-18.14]	0.29
Sex male, n (%)	12 (80.0)		12 (70.6)	0.69
Total IgE (UI/L)	251 [161.50-1358]		59.60 [0.53-402.50]	**0.01**
Sensitization to food and/or respiratory allergens, n (%)	15/16 (94)		6/11 (55)	**0.03**
Symptoms				
Food refusal, n (%)	8 (47)		4 (27)	0.43
Growth retardation, n (%)	3 (18)		1 (1)	0.58
Reflux, n (%)	5 (29)		10 (67)	0.13
Chronic vomiting, n (%)	5 (29)		2 (13)	0.37
Food impaction, n (%)	14 (82)		3 (20)	**0.02**
Dysphagia, n (%)	12 (71)		5 (33)	0.13
Macroscopic findings				
Erythema, n (%)	9 (53)		3 (20)	**0.08**
Furrows, n (%)	4 (24)		1 (7)	0.34
Rings, n (%)	2 (12)		1 (7)	1
Microscopic findings				
Number eosinophils/HPF -upper third	34.00 [13.25-47.00]		0	**< 0.01**
Number eosinophils/HPF - middle third	30.00 [14.25-51.00]		0	**< 0.01**
Number eosinophils/HPF - lower third	36.50 [30.00-68.50]		0	**< 0.01**

BMI, body-mass index; HPF, high-power field. Clinical data were collected at inclusion. For ethical reason, information related to ethnicity were not reported. Biological and cellular measurements were performed in hospital departments of biology and pathology, repsectively. Statistical analysis: groups were compared using the Mann Whitney test or Fisher’s exact test. Bold values are statistically significant (P < 0.05).

### 3.2 Transcriptomic signature of EoE in biopsies from French children

**Figure 1:**
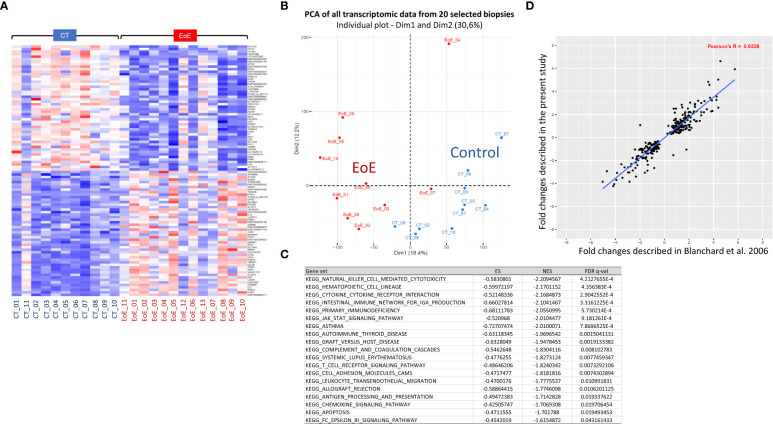
Transcriptomic signature of EoE in children from a French cohort. **(A)** Heatmap of the 50 most significantly upregulated (red) or downregulated (blue) transcripts obtained from biopsies of 11 CT and 13 EoE patients. Expression values are centered on the mean. The magnitude of the gene changes is proportional to the darkness of the color. Each column represents a separate individual and each line a DE gene. **(B)** Non-supervised multivariate analysis (PCA) of all transcriptomic data obtained from the 20 selected biopsies (CT: blue, n = 10; EoE: red, n = 10), showing natural separation of EoE *versus* controls. **(C)** Significantly enriched pathways in EoE patients (n = 10) relative to controls (n = 10), identified through GSEA and considering all analyzed genes. The 19 MSigDB gene sets that were enriched by a FDR q-value < 0.05 are shown. **(D)**. Correlation of the fold changes of 300 DE genes described in (6) (x-axis) compared to our study (y-axis). For functionally organized pathway network identified with ClueGO using the 4,767 identified DE genes, see **Data Sheet 2**.

Among the relevant transcriptomic data obtained from 24 biopsies, gene set enrichment analysis (GSEA) highlighted eighteen significantly enriched pathways in biopsies from EoE patients relative to control subjects, mainly related to immune responses. A clear EoE transcriptomic profile was evidenced when considering the highest differentially expressed (DE) genes (**Figure 1A**). However, the transcriptomic profiles appeared as inverted for biopsies from one CT (CT_11) and three EoE patients (EoE_11 to 13). For these EoE patients, macroscopic changes were observed only in restricted areas of the oesophagus during endoscopy: biopsies analysed for the transcriptome may then have been obtained outside of the pathological areas, reflecting the patchiness of EoE and in line with (32). We then excluded these four samples and compared further the gene expression for biopsies from 10 EoE *vs* 10 CT subjects. We observed a clear separation of the two groups in non-supervised multivariate analysis (PCA, **Figure 1B**), and found 4,767 DE genes among the 57,409 genes/lncRNAs analysed (8.3%, DE genes provided in **Supplementary Table 1**). Within the top downregulated genes, we evidenced various genes involved in (oesophagus) epithelial cell function and barrier integrity: serine peptidase inhibitor Kazal type 8 (SPINK8) and 7 (SPINK7), surfactant associated 2 (SFTA2), epithelial mitogen (EPGN), transglutaminase 3 (TGM3), Secreted LY6/PLAUR domain containing 1 (SLURP1), endonuclease poly(U) specific (ENDOU), mucus proteins (e.g. MUC22, MUC21). Within the top upregulated genes, most were involved in innate and adaptive immunity. This was confirmed by the nineteen enriched pathways evidenced through GSEA (**Figure 1C**), and the 90 enriched canonical pathways identified thanks to complementary IPA (**Supplementary Table 2**). Functionally organized pathway network further showed the major involvement of both innate and adaptive immune networks in EoE (**Data Sheet 2**; **Supplementary Table 3**). This later analysis also evidenced involvement of pathways related to perception of chemical stimulus, cornification, apoptosis, cell organization, migration, and even lipid metabolism.

Interestingly, we found a strong correlation between the fold changes identified in our study and those of Blanchard and collaborators (6) (**Figure 1D**, ρ=0.93). The same top upregulated genes were highlighted, such as TNFα-induced protein 6 (TNFAIP6), CCL26 (eotaxin3), arachidonate 15-lipoxygenase (ALOX15), Periostin (POSTN), and carboxypeptidase-3 (CPA3) [**Supplementary Table 1**, (6)]. Of the 68 DE genes associated with EoE in American cohorts (7), 78% were also found in our list of DE genes, once again with comparable fold changes (not shown).

### 3.3 Cellular and soluble immune constituents in oesophageal biopsies differentiate children with EoE from control subjects.

**Figure 2:**
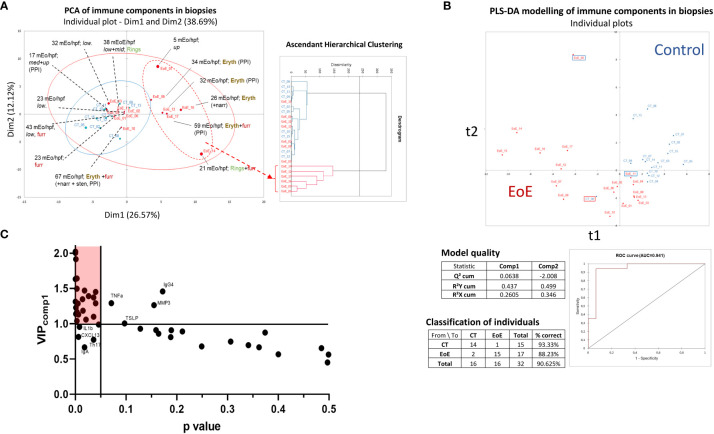
Non-supervised and supervised analysis of cellular and soluble immune constituents in oesophageal biopsies. **(A)** Non-supervised ACP (left) and AHC (right) analysis of all data obtained from EoE patients (red, n = 13 of 17; four have to be excluded from ACP due to missing values) and CT (blue, n = 10 of 15; five excluded due to missing values). Endoscopy observations (erythema: eryth, furrows: furr, rings, narrowing and/or stenosis) and mean eosinophil counts (mEo/hpf) obtained from the three analysed biopsies (upper, middle, and lower third) are indicated for each EoE patient. For some patients, EoE was restricted to the upper (up), middle (mid), and/or lower (low) third part of the oesophagus as indicated. **(B)** PLS-DA modelling constructed with all immune data available (missing values are ignored) from the 17 EoE patients (red) and 15 controls (blue), and model characteristics. Misclassified EoE patients (n=2) and CT (n=1) are shown using framed boxes in the graph. **(C)** Graph of the VIP values obtained on the first component of PLS-DA modelling (VIP_comp1_) x P values obtained following Mann Whitney tests (p < 0.05, without post-test correction) and selection of the significant and discriminant variables (red shaded area). Some named components highly contributed in PLS-DA modelling but showed high P value (upper right) whereas other poorly contributed in PLS-DA modelling but showed P values <0.05 (lower left).

As biopsies and data acquisition methods used are independent for transcriptomics and other analysis, we analysed soluble and cellular immune constituents measured in biopsies from all the patients, i.e. even those excluded from transcriptomic analysis. First, we observed that, among children with EoE, the mean number of eosinophils significantly correlated with ILC count and with concentrations of IgE, CCL22, CCL13, IL-16 and CXCL13 (Spearman correlation, ρ > 0.6; p < 0.05). Then, non-supervised PCA of all cellular and soluble immune constituents measured in biopsies was performed. We did not evidenced a clear separation of children with EoE from control patients using PCA (**Figure 2A** *, left*). However, children with EoE dysplaying eosinophilia on a limited part of the oesophagus had cellular and soluble immune constituent profiles that were close and almost overlapping with those of controls, whereas most of the children with EoE with erythema and eosinophilia in all of the oesophagus clustered separately, as also highlighted in the AHC graph (**Figure 2A** *, right*).

Supervised multivariate modelling of all cellular and soluble immune constituents with EoE status as the explicative variable allowed the construction of a model with good predictive value (**Figure 2B**). Three children were misclassified; one child with EoE was at the interface of the EoE and CT subjects, as already observed for its transcriptomic signature (EoE_11, **Figure 1A**). The other two misclassified samples corresponded to samples with missing data for all soluble constituents (CT_06 and EoE_05), which may affect their classification.

**Figure 3:**
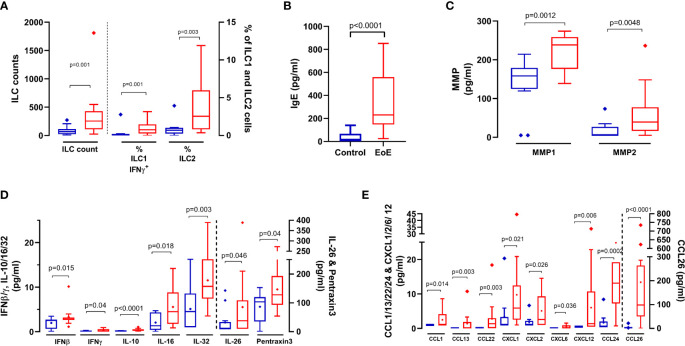
Cellular and soluble components in biopsies that significantly (p < 0.05) discriminate (VIP > 1) between EoE patients (red bars) and controls (blue bars): **(A)** ILC absolute counts, and frequencies of ILC1-IFNγ^+^ and ILC2. ILC1 and ILC2 were identified within live FSC^low^CD45^+^ singlet cells as lin^-^CD127^+^ populations, and then depending on intracellular Tbet and GATA3 expression, respectively. Activated ILC1 were further identified as IFNγ^+^ within ILC1 cells. Activated ILC1 and ILC2 are expressed as % of live FSC^low^CD45^+^ singlet cells. Total IgE **(B)**, Matrix Metalloprotease 1 and 2 (MMP1/2, **(C)**, cytokines **(D)** and chemokines **(E)** concentrations in supernatants obtained from biopsies. P values obtained through Mann-Whitney tests are indicated.

Orthogonal PLS-DA modelling and univariate analysis were then combined and the most discriminant and significant variables that distinguished children with EoE from control subjects were identified using model-calculated variable-important-in-projection values on component 1 of PLS-DA (VIP_comp1_ >1) and a P value <0.05 **(** **Figure 2C**). We then evidenced higher absolute numbers of ILCs and higher frequency of ILC2s and activated ILC1s (ILC1-IFNγ^+^) in biopsies from children with EoE compared to CT (**Figure 3A**). Higher concentrations of IgE (**Figure 3B**), metalloproteases (MMP1 and MMP2, **Figure 3C**), various cytokines (IFNβ, IFNγ, IL-10, IL-16, IL-26, IL-32 and pentraxine-3, **Figure 3D**) and chemokines [mostly eotaxins CCL24 and CCL26, and CCL1, CCL13, CCL22, CXCL1, CXCL2, CXCL6, CXCL12 (**Figure 3E**)] were also found in biopsies from EoE patients than in CT subjects. Additionally, certain components highly contributed in the PLS-DA modelling but showed high P values (TLSP, TNFα, IgG4, and MMP3), whereas other poorly contributed to the PLS-DA modelling (VIP_comp1_ <1 and VIP_comp2_ <1) but showed P values < 0.05 (IL-1β, CXCL13, IgA, and frequency of Th17 cells) (**Figure 2C**); the univariate graphs of the corresponding constituents are shown in **Supplementary Figure 1**.

The number of eosinophils and most of the EoE-specific constituents previously identified were unaffected by PPI medication, except ILC2 frequency and MMP1 concentrations (data not shown and **Supplementary Figures 2A-D**). Among EoE patients, the only local constituent significantly affected by PPIs was sTNFR2 (p=0.021, not shown).

### 3.4 Signature of EoE in periphery

#### 3.4.1 Cellular and soluble immune constituents in blood

**Figure 4:**
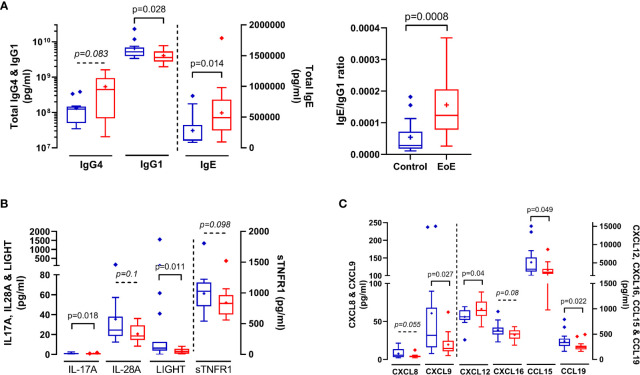
Immune soluble components in plasma that significantly (p < 0.05) or trend (0.05 < p < 0.1) to discriminate (VIP > 1) between EoE patients (red bars) and controls (blue bars): Total IgE, IgG1 and IgG4 **(A)**, cytokines **(B)** and chemokines **(C)** concentrations in plasma. IgE/IgG1 ratio is also shown in **(A)** P values obtained through Mann-Whitney tests are indicated.

Interestingly, cellular and soluble immune constituents assessed in oesophagus and blood were not correlated (not shown). Non-supervised PCA of all soluble and cellular constituents in blood (126 variables) did not show outliers nor natural clustering of patients (not shown), but PLS-DA modelling allowed very good classification of patients (AUC=1; **Supplementary Figure 3A**). Within the discriminant variables (VIP>1), we could not identify significant cellular constituents (p<0.05); only a trend in the increase of circulating activated Th2 cells (Th2-IL13^+^) was observed in EoE patients compared to CT (p=0.06). We then performed an additional supervised analysis integrating only the soluble plasma constituents in the modelling. PLS-DA modelling was still very good at classifying children with EoE (AUC=0.988) and combining PLS-DA and univariate analysis allowed the identification of a EoE signature in blood (**Supplementary Figure 3B**). Compared to CT, children with EoE had significantly higher concentrations of total IgE and significantly lower concentrations of total IgG1, leading to a significant increase in the IgE/IgG1 ratio in EoE (**Figure 4A**). The concentrations of all other soluble constituents that significantly discriminated EoE and controls were decreased in EoE compared to CT, except CXCL12 (**Figure 4B, C**).

#### 3.4.2 EoE metabolomics signature in plasma

Thanks to untargeted mass spectrometry-based metabolomics and an in-house database, we annotated 125 metabolites in plasma (**Supplementary Table 4**). PCA of all identified metabolites did not show a clear separation between children with EoE and control subjects, but non-supervised clustering showed enrichment in a separate cluster of some children with EoE, 5 out 6 not receiving PPI (**Supplementary Figure 4A**). Supervised PLS-DA with EoE status as the explicative variable allowed construction of a model with acceptable quality (**Supplementary Figure 4B**). Two controls (CT_09 and CT_11) and one EoE patient (EoE_05) were misclassified. Interestingly, this later EoE patient was also misclassified when modelling immune data obtained locally (**Figure 2**). When combining univariate and multivariate analysis, we found several discriminant metabolites (**Supplementary Figures 4C**), with theophylline being the most affected quantitatively (fold change = 3.8). However, PPIs can interfere with theophylline metabolism (33) and can influence the concentration of certain metabolites regardless of EoE status (16). We thus performed analysis of the metabolomic signature irrespective of EoE status but depending on PPI use, i.e. compared children receiving PPI (n=10, 3 CT and 7 EoE) *versus* those not receiving PPI (non-PPI n=17, 8 controls and 9 EoE). Although no clear separation of PPI/non-PPI groups was observed using unsupervised analysis (i.e. PCA), supervised analysis allowed construction of a very good model (100% specificity and 90% sensitivity, AUC=1; R²Y_cum_=0.738; R²X_cum_=0.269), and identified two significant (p<0.05) and very discriminant (VIP>1.8) metabolites (glycochenodeoxycholic-acid/glycodeoxycholate, Indoleacetic acid). Others highly discriminant (VIP>1.5) metabolites that trend to be significant (0.05<p<0.1) were highlighted (glycodeoxycholate/glycoursodeoxycholic acid/glycochenodeoxycholic acid, L-cysteine S-sulfate, glycocholic-acid, histidine, theophylline/paraxanthine) (not shown).

**Figure 5:**
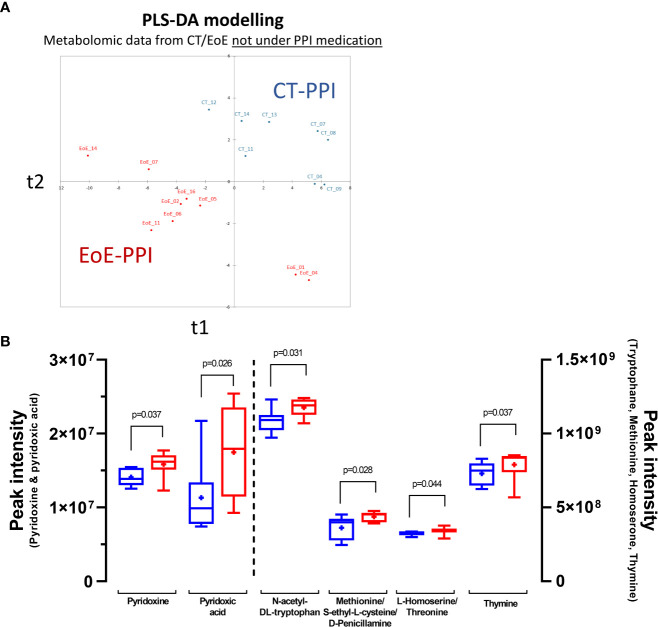
Discrimination of EoE *versus* control based on plasma metabolome: **(A)** Graph of individuals after PLS-DA modelling of metabolomics data from EoE patients not under medication (EoE-PPI: n=9; red) *versus* that from CT not under PPI medication (CT-PPI: n=8; blue). Model characteristics: R²Ycum=0.905, R²Xcum =0.333, AUC=1, 100% sensitivity and specificity. **(B)** Discriminant metabolites showing significant differences when comparing EoE patients (red) to controls (blue).

As PPI medication interfere with our metabolomics signature, notably affecting bile acids metabolism, we conducted new analyses comparing all controls and EoE patients not using PPIs (CT-PPI: n=8; EoE-PPI: n=9). PLS-DA allowed construction of a highly predictive model (model characteristics R²Ycum=0.905, R²Xcum =0.333, AUC=1, 100% sensitivity and specificity; **Figure 5A**). PLS-DA combined with Mann-Whitney tests highlighted discriminant and significant metabolites (**Figure 5B** *):* pyridoxic acid, pyridoxine, methionine/S-ethyl-L-cysteine/D-penicillamine, N-acetyl-DL-tryptophan, L-homoserine/threonine and thymine. Interestingly, the levels of all these metabolites were increased in EoE patients. Others discriminant metabolites (VIP_comp1_ or VIP_comp2_>1.2) also trend (0.05<p<0.1) to be higher in EoE-PPI patients compared to CT-PPI (valine, hippuric-acid, indoleacetic acid, 2-O-methylguanosine/N2-methylguanosine, L-pyroglutamic acid/D-pyroglutamic acid, serine, 3-amino-3-(4-hydroxyphenyl)propanoic/tyrosine/L-threo-3-phenylserine; not shown). Interestingly, some of these metabolites were already highlighted when analysing all patients (i.e. irrespective of PPI medication, **Supplementary Figure 4**; e.g. pyridoxine, hippuric acid, L-homoserine/threonine). Additionnally, pyridoxine, L-homoserine/threonine and 3-amino-3-(4-hydroxyphenyl)propanoic/tyrosine/L-threo-3-phenylserine were also highly discriminant and significant when performing supervised multivariate and univariate analysis of CT-PPI *versus* all EoE patients (not considering PPI use; n=16) (not shown).

### 3.5 Multi-dataset integration

**Figure 6:**
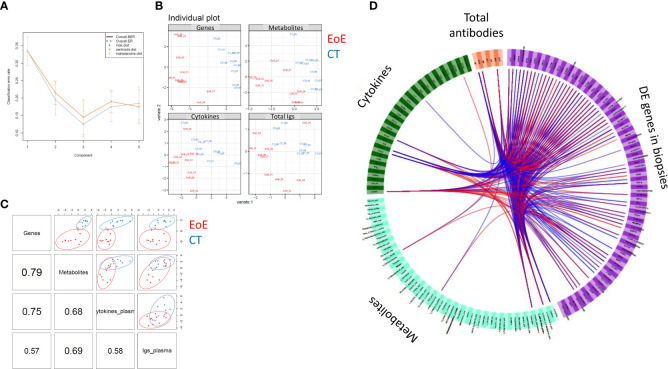
Association between circulating cytokine, antibody, and metabolite levels and local EoE transcriptomic signature. To obtain a statistically robust model, 341 genes were selected, corresponding to the highest and most robust DE expressed genes between EoE patients and controls (|FC| > 1.5, p < 0.01; n = 10 per group, corresponding to individuals with a clear transcriptomics EoE signature and no missing data). All cytokines, antibodies, and metabolites (full names in **Supplementary Table 4**) measured in plasma were considered and all data were scaled before integration. **(A)** Diagnostic test: a three-component model was selected based on the total error (ER) and balanced error rate (BER). **(B)** The individual plots for each dataset (transcriptomics, metabolomics, cytokines and antibodies) showed good separation of EoE patients (red) from controls (blue). **(C)** Pairwise correlations among the different datasets and corresponding distribution of the individuals [same colour code as in **(B)**]. **(D)** Circos plot showing all variables selected by the final DIABLO model for each block. Associations higher than >|0.7| are indicated with blue (negative) or red (positive) lines. Please see **Data Sheet 1** for better resolution.

Finally, as a proof of concept, we tried to perform multi-block association analysis to reveal potential correlation between the local EoE transcriptomic signature and the plasma levels of cytokines, antibodies, and metabolites. This multi-block analysis was performed using the data from 10 CT and 10 EoE patients selected for their EoE transcriptomics signature and who had no missing values in the other datasets. Thirty to 40% of individuals were under PPI medication in both groups. A robust three-component model was obtained (**Figure 6A**) and individual plots for each block showed good separation of EoE patients and controls (**Figure 6B**). Globally, we found a strong correlation between locally DE genes and plasma metabolites (ρ= 0.79) and plasma cytokines (ρ=0.75), whereas the correlation with the levels of total antibodies levels was lower (ρ= 0.57). Additionally, correlation between circulating metabolites, cytokines and total antibodies were lower, ranging between ρ= 0.58 and ρ= 0.68 (**Figure 6C**). Among all the variables selected by the final DIABLO model in each block, we previously identified several as being EoE-specific (IgE, IgG4, CXCL12, pyridoxic acid, hippuric acid, L-homoserine/threonine,…– **Data Sheet 1**). However, in CT and EoE individuals considered for multi-block integration, the metabolites showing the strongest associations with DE genes (ρ >|0.7|) were alanine, acetyl-L-carnitin, hexanoylcarnitine, gamma-butyrolactone, 3-hydroxybutyric acid and alanine. Despite the most significant and discriminant metabolites evidenced in **Supplementary Figure 4** (i.e. considering all CT and EoE, independently of PPI medication) were not highlighted in multi-block analysis based on DE gene, some showing trends were common (i.e. 3-hydroxybutyric acid, gamma-butyrolactone, hexanoylcarnitine and alanine). Plasma sTNFR1 was the cytokine that showed the strongest positive association with DE genes, and IgE and IgG4 levels were strongly associated with numerous metabolites and DE genes, consistent with **Figures 4A, B**. We also observed strong associations between DE genes and the levels of chemokines such as CXCL9, CXCL10, and CCL19.

## 4 Discussion

In this study, we described the local transcriptomic signature of French children with EoE and identified immune constituents involved in the pathogenesis of EoE by a comprehensive immune analysis of biopsies. Moreover, we provided preliminary results to envisage the identification of an EoE signature in plasma.

Firstly, we evidenced the same global transcriptomic signature in our paediatric population from France as in patients from the US, with a very high correlation between the fold changes of DE genes between studies. Our transcriptomic analysis further supports that the EoE transcriptome signature is conserved regardless of the countries, sex, age, PPI use and allergic status (5–7, 34). Transcriptomic analysis and network visualization of DE genes further highlighted the dysregulation of major immune processes, both innate and adaptive. It also identified other dysregulated pathways, notably major downregulation of genes involved in epithelial cell and barrier functions (SPINK6/7/8, SFTA2, EPGN, TGM3, SLURP1, ENDOU, MUC22, MUC21), in line with previous studies (35–37). The involvement of IL-20 subfamily in this epithelial barrier impairment was less evident than that observed recently in adults (37). Indeed, and despite we did evidence a high dysregulation of MAPK cascade pathway (**Supplementary Table 3**) and moderate upregulation of IL-19 gene, we did not evidence dysregulation of expression of genes coding for IL-20 and IL-24, nor for filaggrin or tight junctions proteins. Whatever, EoE is clearly associated with local epithelial barrier dysfunction (38), as for other allergic diseases (39). In addition to downregulation of genes related to epithelial barrier function, it is worth noting that functionally organized pathway network analysis highlighted dysregulation of pathways related to perception of chemical stimulus in EoE. In line with these results, a recent study using cell culture and animal models evidenced that synthetic surfactant, such as detergent found in household products, decreased epithelial barrier integrity, induced IL-33, MMP and eotaxin productions, and promoted both epithelial hyperplasia and tissue eosinophilia (40). The role of environmental chemicals in the initiation / perpetuation of EoE in children thus clearly warrant further investigations. Conversely, and considering the critical role of vitamin D in EoE pathogenesis (35), VDR gene was rather increased in biopsies from our EoE patients (log_2_[FC] = 1.557, p=0.003).

In addition to immune dysregulation evidenced through transcriptomics, our study provides the more comprehensive analysis of both cellular and soluble immune components in oesophageal biopsies from children with EoE *versus* control subjects. Using a biopsy explant culture, Sayej et al (10) detected 24 cytokines and described a panel of 13 cytokines that may contribute to EoE pathogenesis. In line with our results, they evidenced increase in IL-10, TNFα and MMP3 production by EoE explant biopsies. Conversely, they did not evidence differences in IFNγ or TNFβ concentrations, and we did not confirm the increased levels of IL-6, IL-8, CCL2 (MCP1) or sTNFR2 they observed. Such differences may relies on the different methodologies used (explant culture *versus* extraction from fresh biopsies), but also on confounding factor such as PPI medication as we shown in the present study for sTFNR2.

Our results support the involvement of local T2 inflammation in the pathogenesis of EoE. Indeed, we observed an elevated concentration of total IgE in biopsy that strongly correlated with the number of eosinophils and ILC2s. We also evidenced elevated concentrations of CCL24 (Eotaxin-2) and CCL26 (Eotaxin-3) and CCL11, as previously observed (6, 7, 34), and a trend towards higher concentrations of the Th2-related alarmin TSLP. Despite we did not detect increased mRNA IgE levels, concentrations of IgE in biopsies did not correlate with that assayed in plasma (not shown); IgE antibodies may then result, at least partially, from local production (11). Increased concentrations of IgE was not evidenced in other study including paediatric EoE subjects, which rather highlighted increase of IgG4 (9). Th2 cytokines, notably IL-13, are also involved in EoE pathogenesis (41, 42). Despite elevated expression of IL-13 RNA transcripts (log_2_[FC]=2.99), and to a lesser extent of that of IL-5, in line with (10, 32, 34), Th2 cytokines were not detectable at the protein level in our study. As few numbers of lymphocytes expressing IL-13 or IL-5 mRNA were detected in biopsies from children with EoE (12), these results may suggest that Th2-cytokine expression and action may be limited and restricted to immunological synapses. Interestingly, we observed comparable upregulation of *IL5rα* [in line with (32)] and *IL-13Rα2* transcripts in biopsies (log_2_[FC]=1.9627 and 1.941, respectively), whose involvement in EoE pathogenesis would require further attention. Conversely, we observed a decreased expression of *IL-13Rα1* (log_2_[FC]=-0.4969) and no change in *IL-4Rα* expression, thus not in line with the recently suggested major role of IL-13 *via* type 2 IL-4 receptor in EoE (41).

In parallel with T2 markers, high local inflammation was confirmed in the biopsies from EoE patients, with increased levels of IFNβ, IL-1β, CCL1/13/22, CXCL1/2/6/12/13, and MMP1/2, for which some were already shown to be upregulated at the mRNA level (6). We also found increased concentration of IL-16, a cytokine linked to inflammatory processes, such as in asthma, in which its levels correlated with the number of infiltrating CD4^+^ cells (43, 44). On the other side, in addition to IL-10, we observed a significant increased concentration of IL-26, a member of the IL-10 family that has a role in tissue remodelling and wound healing. IL-26 mRNA has been shown to be upregulated in activated Th2 cells isolated from EoE biopsies (12). Upregulation of this cytokine has also been found in autoimmune inflammatory diseases, such as Crohn’s disease or rheumatoid arthritis, and it can be produced by Th17 cells (45), a cell type that was enriched in our biopsies. In summary, increased frequency of various Th2 markers in biopsy, but also of Th17 and ILC1-IFNγ+ cells and of a large panel of soluble factors underlined the complexity of EoE pathology, with involvement of various immune actors far beyond a simplistic T2 immune response, as recently observed for severe asthma (25). As observed for transcriptomics, our immune signature was almost not affected by PPI, which only affected ILC2 frequency, MMP1 concentrations and, within EoE patients, sTNFR2. This can explain PPI therapeutic failure in our patients.

Given the burden of repeated endoscopy procedures for the diagnosis and follow-up of children with EoE, the translation of research findings on biopsies into a viable serum test for the presence and/or severity of EoE would be of enormous value. Using a 29-plex cytokines panel, Blanchard et al concluded EoE is not characterized by a reproducible and consistent dysregulation of blood cytokines levels (32). Accordingly, in a large prospective study, serum samples from adult EoE patients were analysed for a number of soluble immune factors; no difference between EoE patients and controls were found and the measured markers, alone or in combination, had little diagnostic value (14). Using a more comprehensive and untargeted approach, we first observed that almost all immune markers in affected tissue and in periphery were not correlated, as we also observed for severe asthma (25). Such an approach may then be valuable to identify a signature in periphery but not for a better understanding of pathophysiological mechanisms. Secondly, thanks to our comprehensive approach combining 79 detectable soluble immune components in plasma, we evidenced an EoE immune signature in the blood. Compared to CT, we observed higher concentrations of IgE and CXCL12 associated with lower concentrations of a number of chemokines (CXCL8, CXCL9, CXCL16, CCL15, CCL19), cytokines (IL-17A, IL-28A, LIGHT, sTNFR1) and IgG1 in the blood of EoE patients. Plasma CXCL12 seems not increased in other eosinophil related pathology such as asthma (25), suggesting that it may be specific for EoE. Conversely, we did not observe increased concentrations of the IL-20 subfamily cytokines in plasma from our EoE children, on the opposite to recent observations in adults (37), nor increased concentrations of IL-4, IL-5, IL-13, IL-6, IL-8 or IL-1α as observed but not reproduced in (32).

Despite the interest and originality of these results, the lack/small number of positive markers for EoE may not be sufficient to develop efficient diagnostic tools with high sensitivity and specificity. However, non-targeted metabolomics allowed us to identify a metabolomics signature for EoE in plasma. Our metabolomics approach did not allow detecting more lipophilic compounds such as vitamin D, which serum levels were recently shown to be inversely correlated with degree of histopathological changes associated with EoE (35). However, a number of metabolites were significantly elevated in plasma from EoE patients independently of PPI medication, notably the vitamin B6-related compounds pyridoxine and 4-pyridoxic acid (the major excretory form of vitamin B6), and other metabolites including N-acetyl-DL-tryptophan. Tryptophan metabolism has been previously shown to be associated with atopic disorders (46), with increased in tryptophan levels associated with eosinophilic inflammation and asthma symptom scores (47). The EoE signature related to Vitamin B6 is of particular interest and has not been yet described. A deficiency of Vitamin B6 is often associated with inflammatory diseases. Vitamin B6 contributes to intestinal immune regulation through the metabolism of the lipid mediator sphingosine-1-phosphate (S1P) (48, 49), a metabolite also involves in Th1/Treg homeostasis (50). However, we instead found increased concentrations of plasma pyridoxine and pyridoxic acid in EoE patients, which were not associated with differences in S1P levels (not shown). We also observed a positive correlation between pyridoxine levels and those of various amino acids (e.g. tryptophan, phenylalanine, methionine, L-cysteine, (iso)leucine, valine, L-Aspartic acid, arginine, proline, and lysine; ρ ranging from 0.364 to 0.692, p<0.05, Spearman, correlation) and a negative one with L-glutamine levels (ρ=-0.367), consistent with the role of Vitamin B6 in amino-acid metabolism. Vitamin B6 is provided by the diet or produced by a number of bacterial species from the microbiota (48), but we found no difference in the diet between EoE patients and controls that could explain such differences. Potential microbiota dysbiosis leading to such Vitamin B6 dysregulation should thus be explored, either as the cause or consequence of EoE. Moreover, given the various roles of Vitamin B family members in immunometabolism and the regulation of T and B cells, as well as that of MAIT cells (51), further assessment of the effect of Vitamin B6 on innate and adaptive immune cells should be conducted in the context of EoE pathogenesis.

Finally, the integration of our data from a sub-cohort showed the correlation of the EoE transcriptomics signature in biopsies with circulating metabolites and immune components. Such data integration between local EoE transcriptomics (i.e. validated EoE local signature) and circulating molecules (metabolites, cytokines, antibodies) further reinforced the concept that a circulating signature can be identified for EoE by combining multi-omics approaches. As a proof of concept, we selected the twelve most EoE-contributing plasma immune components (IgE, IgG4, CXCL12, sTNFR1) and metabolites (pyridoxine, 4-pyridoxic acid, N-acetyl-DL-tryptophan, thymine, 3-hydroxybutyric acid, gamma-butyrolactone, hexanoylcarnitine, alanine) identified through mono- and multi-blocks analysis and performed a PLS-DA modelling, with EoE status as the explicative variable. We were then able to obtain a good predictive model of EoE status (R²Ycum=0.542; R²Xcum=0.403, AUC=0.929; specificity 93.3% and sensitivity 87.5%). PLS-DA modelling with only Pyridoxine, (S)-3-Hydroxybutyric-acid, Gamma-butyrolactone, CXCL12, IgE and IgG4 data already provided a good model (R²Ycum=0.542; R²Xcum=0.403; AUC 0.904), but with quite lower specificity (80%) and sensitivity (81.25%). Such a signature, i.e. a set of EoE biomarkers in plasma, have to be analysed in a larger and independent cohort for validation. It would be of great value for EoE diagnosis and follow up.

In conclusion, our results support the local involvement of an epithelial barrier defect combined with immune dysregulation in EoE, far beyond the simplistic view of T2 dysregulation. Multi-omics approach such as performed in the present study, integrating plasma immune components and metabolites, would allow identifying a signature of EoE in periphery.

## Data availability statement

The datasets presented in this study can be found in online repositories. The names of the repository/repositories and accession number(s) can be found below: GSE184182 (GEO).

## Ethics statement

The studies involving human participants were reviewed and approved by local ethics committee of Necker Hospital CPP Ile de France II (n°2016-06-03). Written informed consent to participate in this study was provided by the participants’ legal guardian/next of kin.

## Author contributions

Authors’ contributions: FCam, GL, and KA-P: designed the research. BG, CDi, FCas, FF, FM, KA-P, MG, ML-M, PB, and TM: performed the research; CDe, FCam and GL: were responsible for patient recruitment or establishing the patient database. EV, KA-P and MM: analyzed the data. FCam, GL, KA-P: wrote the manuscript. All authors contributed to the article and approved the submitted version.

## Supplementary material

Supplementary Table 1 List of genes found to be differentially expressed in biopsies from EoE patients compared to controls. The second column indicates the log2 FC values and the third column the Benjamini-Hochberg-adjusted p-values.

Supplementary Table 2 List of all the significantly enriched canonical pathways according to the IPA analysis. Only the pathways with a -log(p-value) > 2.50 and including at least 10 genes are listed.

Supplementary Table 3 Table listing all the GO terms found by the ClueGO analysis. The same term can be included in several groups. Terms included in groups with a corrected p-value < 10E-10 are higlighted in pale yellow.

Supplementary Table 4 Table listing all the metabolites identified in plasma. Identification levels: **(A)** Based on accurate mass; **(B)** Based on ZIC-pHILIC column retention time similarity with a standard; **(C)** Based on C18 column retention time similarity with a standard.

Supplementary Figure 1 Cellular and soluble components in biopsies that were either significantly different (p < 0.05, Mann Whitney test) or discriminating (VIPPLS-DA >1) between EoE (red) and controls (blue). **(A)** Th17 cell frequencies were identified among live FSC^low^CD45^+^ singlet cells as lin^+^CD4^+^RORγt^+^ cells. Total IgA and IgG4 **(B)** and MMP, cytokine, and chemokine **(C)** concentrations in supernatants obtained from biopsies. P values obtained following Mann-Whitney tests are indicated.

Supplementary Figure 2 Cellular and soluble components in biopsies depending on PPI use. EoE counts **(A)**, ILC frequencies **(B)**, and total IgE **(C)** and cytokine **(D)** concentrations in biopsies from controls not receiving PPIs (Ct-PPI, n=8, open blue circles) and EoE patients (red symbols) not receiving (EoE-PPI, n=9-10, open circles) or receiving (EoE+PPI, n=6-7, full circles) PPIs. “a” and “b” indicate statistical significance following Kruskall Wallis tests and Dunn’s post-test for multiple comparisons: groups sharing the same letter show no difference. Values obtained for all EoE patients, irrespective of PPI use, are shown as red bars (see Figure 3).

Supplementary Figure 3 Supervised analysis of cellular and soluble **(A)** or only soluble **(B)** immune constituents in the periphery to distinguish EoE from controls. PLS-DA modelling of constituents assayed on blood/plasma from 17 EoE patients (red) and 15 controls (blue). Model characteristics are indicated and the misclassified patient in B is shown using a framed box in the graph. For both A and B, immune constituents that significantly discriminate EoE from controls were selected on the graph plotting the VIP values obtained for the first component of PLS-DA modelling and the P values obtained following Mann Whitney tests (the cut-offs at p < 0.05 (dark red) and 0.05 < p < 0.1 (light red) are shown).

Supplementary Figure 4 Discrimination of EoE (red) versus control (blue) based on the plasma metabolome obtained from all patients. **(A)** Non-supervised ACP and CAH of annotated metabolites. **(B)** PLS-DA modelling of metabolites using the EoE vs control status as the explanatory variable. **(C)** Discriminant metabolites showing a significant increase or a trend towards an increase in EoE patients relative to controls. P values (Mann Whitney testing) and fold changes between EoE and CT are indicated.
