# Supplementary figures and images for: Assessment of local and systemic signature of eosinophilic esophagitis (EoE) in children through multi-omics approaches
Source: Front Immunol. 2023 Mar 15;14:1108895. doi: 10.3389/fimmu.2023.1108895 (PMC10050742; doi:10.3389/fimmu.2023.1108895)

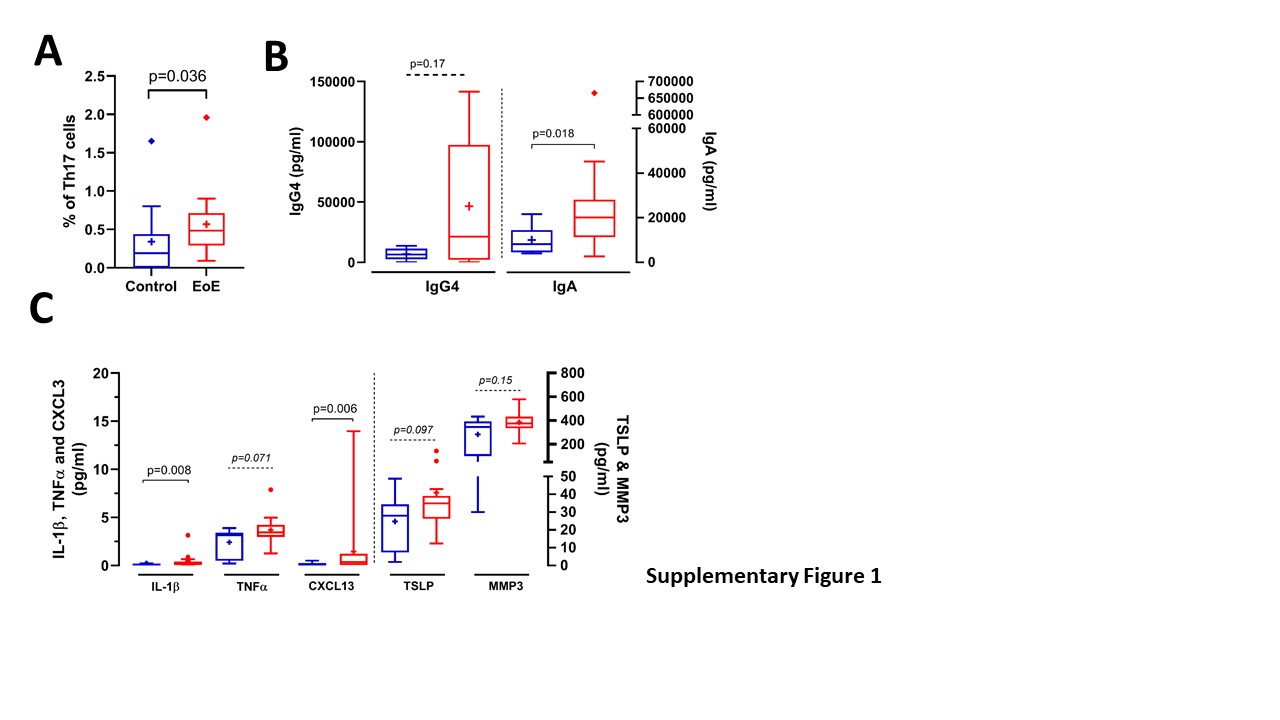

Supplement: Supplementary Figure 1 — Cellular and soluble components in biopsies that were either significantly different (p < 0.05, Mann Whitney test) or discriminating (VIPPLS-DA >1) between EoE (red) and controls (blue). (A) Th17 cell frequencies were identified among live FSClowCD45+ singlet cells as lin+CD4+RORγt+ cells. Total IgA and IgG4 (B) and MMP, cytokine, and chemokine (C) concentrations in supernatants obtained from biopsies. P values obtained following Mann-Whitney tests are indicated. [file Image_1.jpeg]

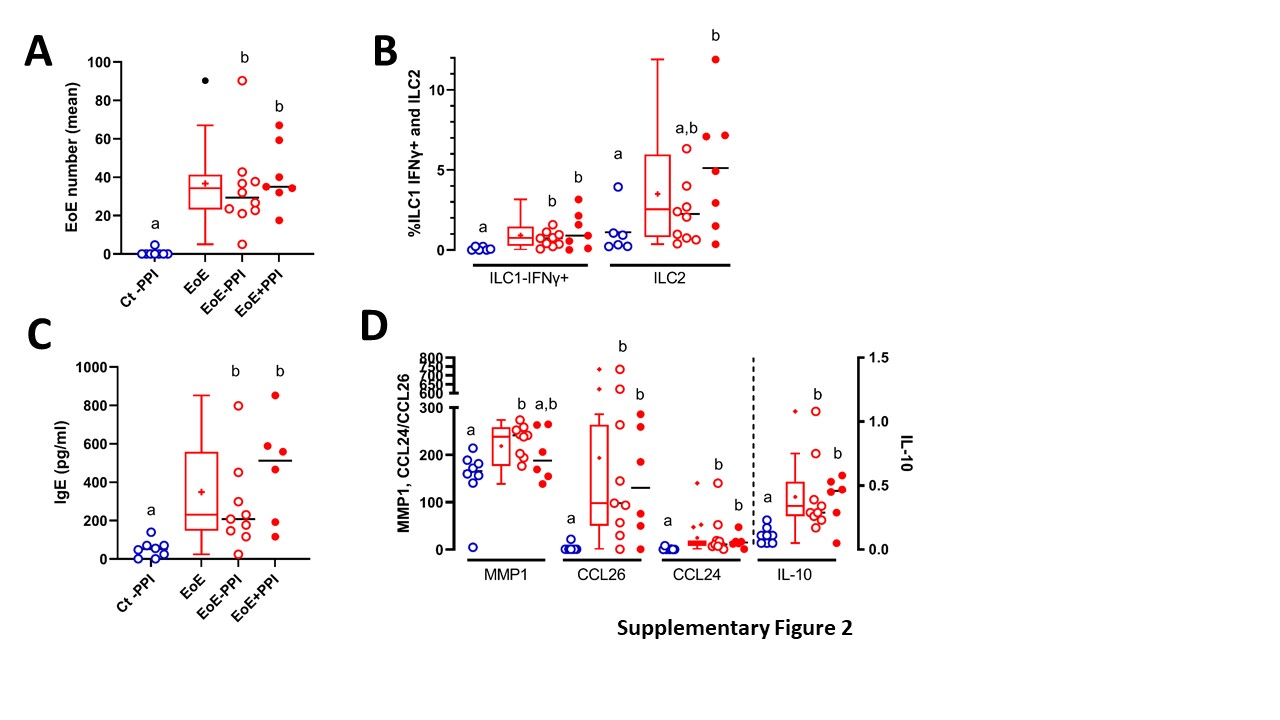

Supplement: Supplementary Figure 2 — Cellular and soluble components in biopsies depending on PPI use. EoE counts (A), ILC frequencies (B), and total IgE (C) and cytokine (D) concentrations in biopsies from controls not receiving PPIs (Ct-PPI, n=8, open blue circles) and EoE patients (red symbols) not receiving (EoE-PPI, n=9-10, open circles) or receiving (EoE+PPI, n=6-7, full circles) PPIs. “a” and “b” indicate statistical significance following Kruskall Wallis tests and Dunn’s post-test for multiple comparisons: groups sharing the same letter show no difference. Values obtained for all EoE patients, irrespective of PPI use, are shown as red bars (see Figure 3). [file Image_2.jpeg]

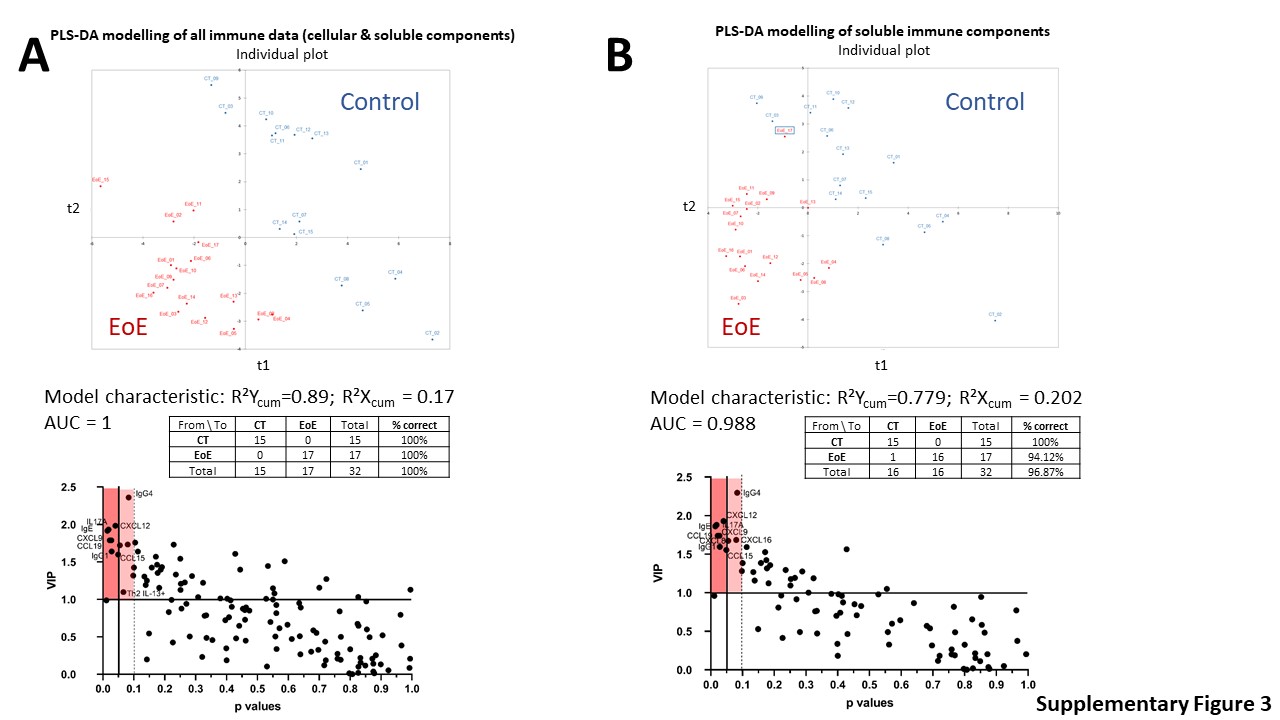

Supplement: Supplementary Figure 3 — Supervised analysis of cellular and soluble (A) or only soluble (B) immune constituents in the periphery to distinguish EoE from controls. PLS-DA modelling of constituents assayed on blood/plasma from 17 EoE patients (red) and 15 controls (blue). Model characteristics are indicated and the misclassified patient in B is shown using a framed box in the graph. For both A and B, immune constituents that significantly discriminate EoE from controls were selected on the graph plotting the VIP values obtained for the first component of PLS-DA modelling and the P values obtained following Mann Whitney tests (the cut-offs at p < 0.05 (dark red) and 0.05 < p < 0.1 (light red) are shown). [file Image_3.jpeg]

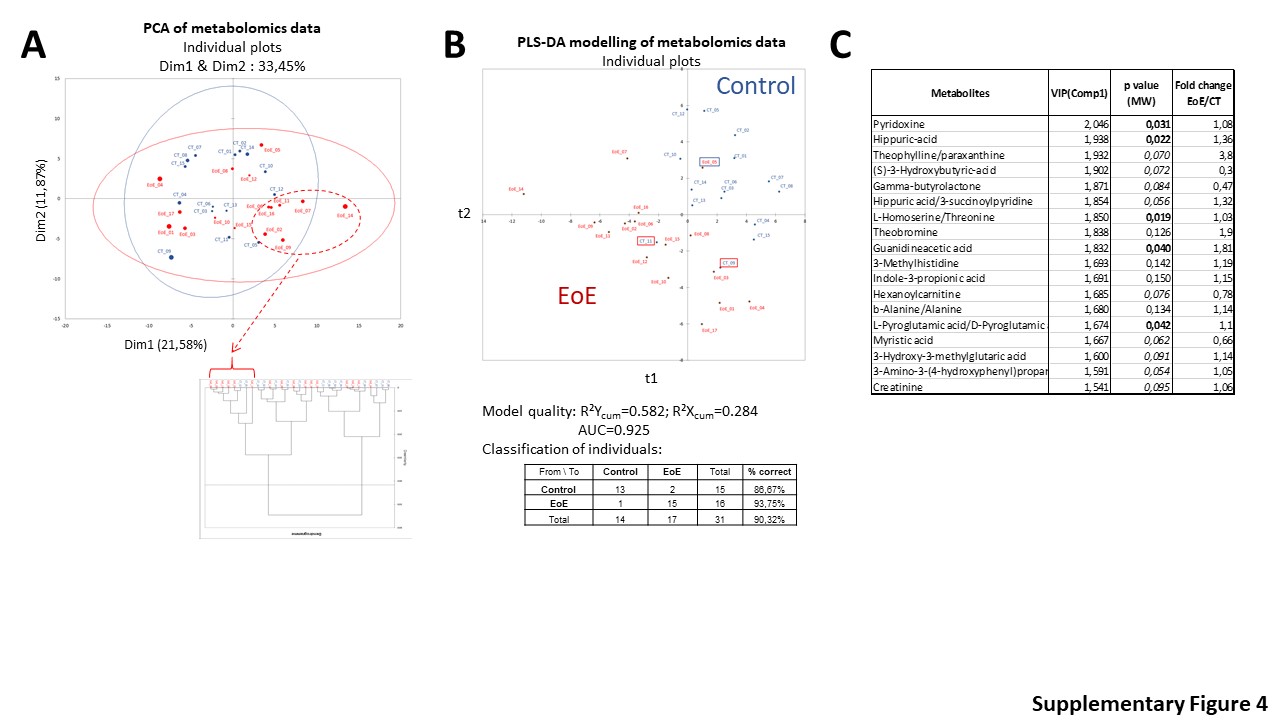

Supplement: Supplementary Figure 4 — Discrimination of EoE (red) versus control (blue) based on the plasma metabolome obtained from all patients. (A) Non-supervised ACP and CAH of annotated metabolites. (B) PLS-DA modelling of metabolites using the EoE vs control status as the explanatory variable. (C) Discriminant metabolites showing a significant increase or a trend towards an increase in EoE patients relative to controls. P values (Mann Whitney testing) and fold changes between EoE and CT are indicated. [file Image_4.jpeg]

Comp 1-2-3

Igs\_plasma

Correlation cut-off  
 $r=0.7$

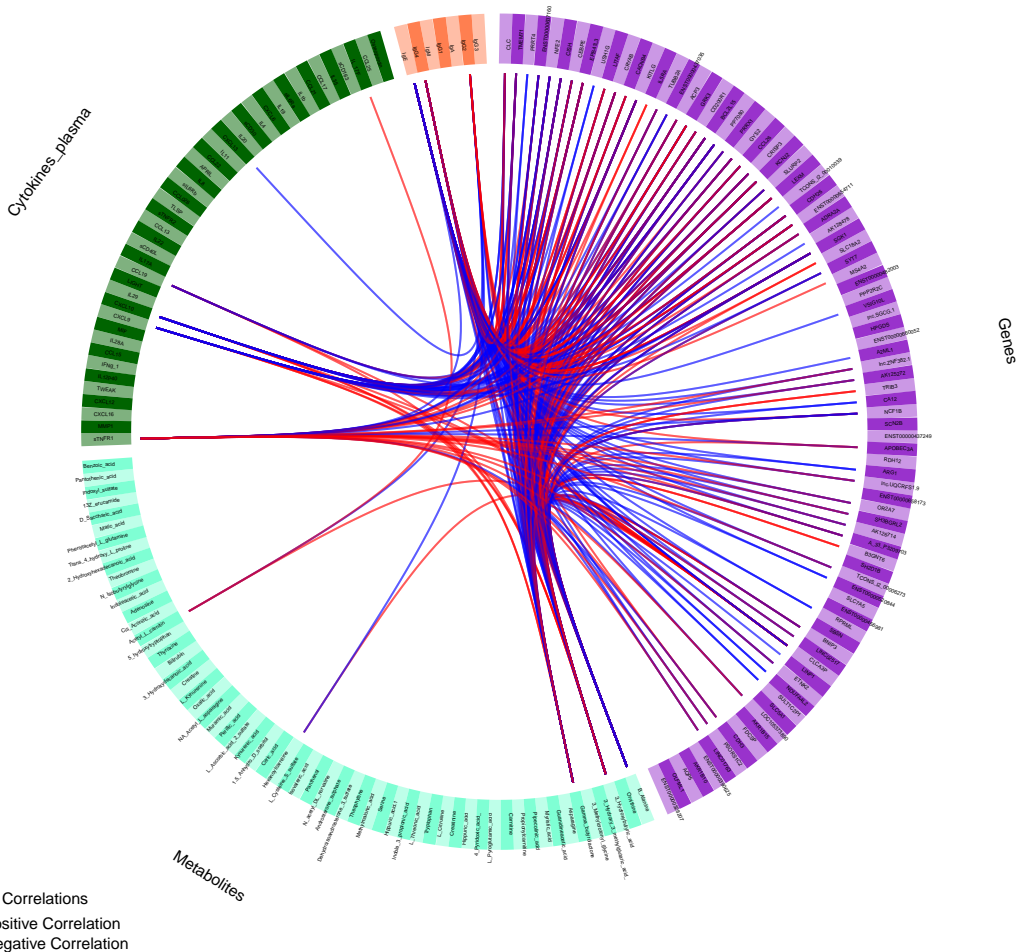

Supplement: Supplementary file 9 [file DataSheet_1.pdf]

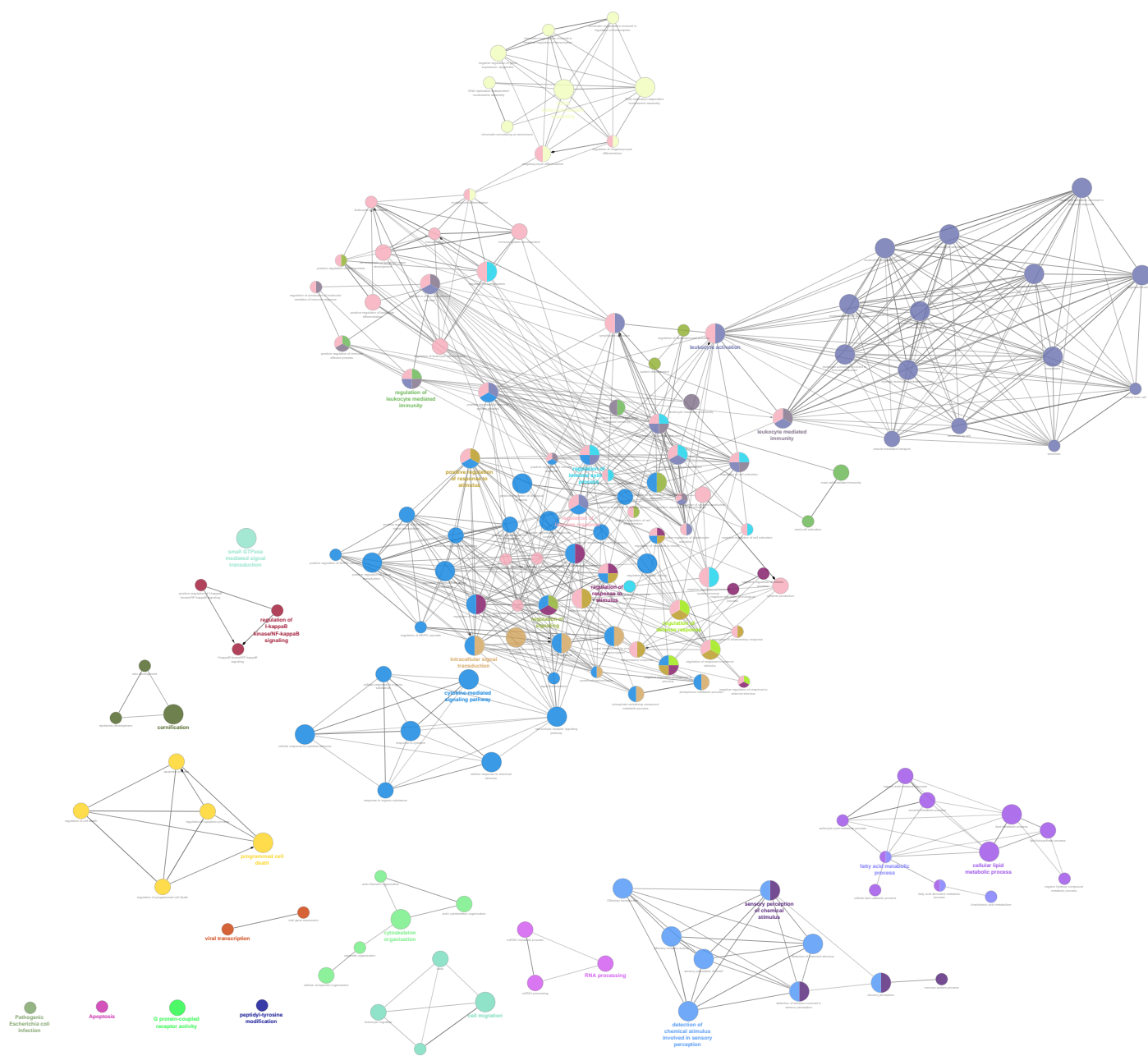

Supplement: Supplementary file 10 [file DataSheet_2.pdf]
